# Microbial characterization of probiotics–Advisory report of the Working Group “8651 Probiotics” of the Belgian Superior Health Council (SHC)

**DOI:** 10.1002/mnfr.201300065

**Published:** 2013-06-25

**Authors:** Geert Huys, Nadine Botteldoorn, Frank Delvigne, Luc De Vuyst, Marc Heyndrickx, Bruno Pot, Jean-Jacques Dubois, Georges Daube

**Affiliations:** 1 Laboratory for Microbiology & BCCM/LMG Bacteria Collection Faculty of Sciences, Ghent UniversityGhent, Belgium; 2 Scientific Service Food-Borne Pathogens, Institute of Public HealthBrussels, Belgium; 3 Unité de bio-industries Gembloux Agro-Bio Tech, Université de LiègeGembloux, Belgium; 4 Research Group of Industrial Microbiology and Food Biotechnology Faculty of Sciences and Bioengineering Sciences, Vrije Universiteit BrusselBrussels, Belgium; 5 Institute for Agricultural and Fisheries Research, Technology & Food Science Unit – Research Area Food SafetyMelle, Belgium; 6 Department of Pathology Bacteriology and Avian Diseases Faculty of Veterinary Medicine, Ghent UniversityGhent, Belgium; 7 Bactéries Lactiques et Immunité des Muqueuses Centre d’Infection et d’Immunité de Lille (CIIL) Institut Pasteur de Lille, University Lille Nord de FranceLille, France; 8 Federal Public Service (FPS) Health, Food Chain Safety and Environment, Belgian Superior Health CouncilBrussels, Belgium; 9 Laboratoire de Microbiologie des Aliments Département des Sciences des Denrées Alimentaires Faculté de Médecine Vétérinaire Université de Liège, Sart-TilmanLiège, Belgium

**Keywords:** Identification, Lactic acid bacteria, Probiotics, Product quality control, Safety

## Abstract

When ingested in sufficient numbers, probiotics are expected to confer one or more proven health benefits on the consumer. Theoretically, the effectiveness of a probiotic food product is the sum of its microbial quality and its functional potential. Whereas the latter may vary much with the body (target) site, delivery mode, human target population, and health benefit envisaged microbial assessment of the probiotic product quality is more straightforward. The range of stakeholders that need to be informed on probiotic quality assessments is extremely broad, including academics, food and biotherapeutic industries, healthcare professionals, competent authorities, consumers, and professional press. In view of the rapidly expanding knowledge on this subject, the Belgian Superior Health Council installed Working Group “8651 Probiotics” to review the state of knowledge regarding the methodologies that make it possible to characterize strains and products with purported probiotic activity. This advisory report covers three main steps in the microbial quality assessment process, i.e. (i) correct species identification and strain-specific typing of bacterial and yeast strains used in probiotic applications, (ii) safety assessment of probiotic strains used for human consumption, and (iii) quality of the final probiotic product in terms of its microbial composition, concentration, stability, authenticity, and labeling.

## 1 Introduction

The word “probiotic” means “for life” (from the Greek *προ βίος*, *pro bios*). The works of Metchnikoff [1] and Tissier [2] were the first to make scientific suggestions concerning the probiotic use of bacteria, even when the word “probiotic” was not coined until 1960, to name substances produced by microorganisms that promoted the growth of other microorganisms [3]. Some other definitions followed, with Fuller [4] being the first pointing out the microbial nature of probiotics by redefining the word “probiotic” as “a live microbial feed supplement that beneficially affects the host animal by improving its intestinal balance”. Havenaar and Huis in’t Veld [5] extended the definition as “a viable mono- or mixed culture of bacteria which, when applied to animal or man, beneficially affects the host by improving the properties of the indigenous flora”. Guarner and Schaafsma [6] gave a more recent definition as “live microorganisms, which when consumed in adequate amounts, confer a health effect on the host”.

According to the report of the joint “Food and Agriculture Organization/World Health Organization (FAO/WHO) Expert Consultation on Evaluation of Health and Nutritional Properties of Probiotics in Food including Powder Milk with Live Lactic Acid Bacteria” [7], probiotics were redefined for the purpose of the meeting as: “Live microorganisms which when administered in adequate amounts confer a health benefit on the host” (FAO/WHO, 2001). Following the FAO/WHO definition, the International Life Sciences Institute [8] and the European Food and Feed Cultures Association [9] have launched similar definitions for a probiotic, namely “a live microbial food ingredient that, when taken up in adequate amounts, confers health benefits on the consumers” and “live microorganisms which, when ingested or locally applied in sufficient numbers, provide the consumer with one or more proven health benefits”, respectively.

The FAO/WHO expert consultation restricted its scope to the discussion of probiotics “as part of the food” and excluded reference to the term biotherapeutic agents and beneficial microorganisms not used in food. However, it is recognized that the definition is sufficiently broad as to encompass a range of probiotic preparations and intentions of use. In this respect, a probiotic can be a food or a dietary supplement, can be used in a drug application (also named a live biotherapeutic) or microbial feed or can be used as genetically modified microorganism (GMM) or live vaccine if administered orally.

For use in food, important criteria for probiotics were documented, in particular that they should not only be capable of surviving passage through the digestive tract, by exhibiting acid and bile tolerance and withstand digestive enzymes, but also have the capability to proliferate in the gut. Probiotics must be able to exert their benefits on the host through growth and/or activity in the human body. Therefore, the ability to remain viable at the target site and to be effective should be verified for each strain.

In 2002, a joint FAO/WHO working group generated Guidelines for the evaluation of Probiotics in Food [10]. It was recommended to officially adopt the above FAO/WHO definition of probiotics and to use and adopt the guidelines in the report of this working group as a prerequisite to call a microbial strain “probiotic”. The minimum requirements needed for probiotic status include:

1. the assessment of strain identity (genus, species, strain level);
2. in vitro tests to screen potential probiotic strains: e.g. resistance to gastric acidity, bile acid and digestive enzymes, antimicrobial activity against potentially pathogenic bacteria, etc.;
3. assessment of safety: requirements for proof that a probiotic strain is safe and without contamination in its delivery form;
4. in vivo studies for substantiation of health effects in the target host.

The working group recommended that information accumulated to show that a strain is a probiotic, including clinical trial evidence, be published in peer-reviewed scientific or medical journals. Also, publication of negative results is encouraged, as to contribute to the totality of the evidence to support probiotic efficacy.

The recommendations of the above FAO/WHO Consultation and the Guidelines of the FAO/WHO working group have been assembled in one document [11] and were presented to two Codex Alimentarius Committees: the Codex Committee on Food Labeling and the Codex Committee on Nutrition and Foods for Special Dietary Uses. It is hoped that the work of the FAO/WHO probiotics groups will be used as a model for future Codex guidelines.

In relation to the substantiation of health effects of probiotics in the target host, it is important to know that in the European Union, health claims should only be authorized for use in the Union after a scientific assessment of the highest possible standard has been carried out by the Panel on Dietetic Products, Nutrition, and Allergies (NDA) of the European Food Safety Authority (EFSA) (Regulation (EC) No. 1924/2006) [12]. Key questions that are addressed by the EFSA NDA panel are:

1. is the food/constituent sufficiently defined and characterized?
2. is the claimed effect sufficiently defined, and is it a beneficial physiological effect?
3. have pertinent human studies been presented to substantiate the claim?

If the outcome of all of the above questions is favorable, the panel weighs the evidence from all the pertinent studies presented, including human, animal, in vitro, and mechanistic studies.

The NDA panel has provided general guidelines on how health claims related to gut and immune function are scientifically evaluated [13]; these guidelines are particularly interesting for probiotics that are usually expected to have this kind of health effects. The guidelines are not intended as an exhaustive list of beneficial effects and acceptable studies/outcome measures, but rather present examples drawn from evaluations already carried out to illustrate the approach of the panel.

Finally, it should be emphasized that besides strain identification and characterization, survival of the passage of the upper gastrointestinal tract, transient adhesion to or interaction with the intestinal epithelium, and colonization of the colon and resistance of the strains toward technological processing and storage are of utmost importance. This underlines the importance of the food matrix and the applied process technology to prepare the food (constituent) and the requirement for stability and survival of the strain until the moment of consumption of the food. This in turn points toward food matrix dependency (including amount of food to be consumed for a proven health effect) and the importance of an adequate amount of live active cells, respectively.

In view of the rapidly expanding knowledge on this subject, this advisory report on the issue of probiotics provides up-to-date information on the state of the art on the microbiological characterization of the concerned live microorganisms (bacteria and yeasts).

## 2 Strain identification and typing

### 2.1 Microbial species used as probiotics for human application

A wide range of product types containing viable or heat-killed microorganisms with probiotic claims are commercially available either as fermented food commodities, foods with probiotic ingredients, or in lyophilized form. In accordance to the widely used FAO/WHO definition of probiotics [7], this chapter will only deal with the identification and typing of live microorganisms used as probiotics for human applications (Table1).

**Table 1:** Most important microorganisms applied in probiotic products for human usea)–c))

Lactic acid bacteria	*Bifidobacterium*	Other bacteria	Yeasts
*Lactobacillus*	*Bf. adolescentis*	*Bacillus*	*Saccharomyces cerevisiae* var. boulardii
*Lb. acidophilus*	*Bf. animalis* subsp*. lactis*	*Bc. cereus*d)	*Saccharomyces* spp.
*Lb. casei/paracasei*	*Bf. bifidum*	*Bc. coagulans*	
*Lb. crispatus*	*Bf. breve*	*Bc. clausii*	
*Lb. fermentum*	*Bf. longum* subsp*. infantis*	*Bc. pumilus*	
*Lb. gallinarum*	*Bf. longum* subsp*. longum*	*Bc. subtilis*	
*Lb*. *gasseri*		*Escherichia coli* Nissle 1917d)	
*Lb*. *johnsonii*		*Propionibacterium*	
*Lb*. *plantarum*		*Pr. acidipropionici*	
*Lb*. *reuteri*		*Pr. freudenreichii* subsp*. shermanii*	
*Lb*. *rhamnosus*		*Pr. jensenii*d)	
*Lb. salivarius*			
*Enterococcus faecium*d)			
*Lactococcus lactis* subsp. *lactis*			
*Leuconostoc*			
*Le. citreum*			
*Le. mesenteroides* subsp. *cremoris*			
*Oenococcus oeni* e)			
*Pediococcus*			
*Pd. acidilactici*			
*Pd. pentosaceus*			
*Sporolactobacillus inulinus*d)			

a) Adapted and updated from [14,15,17].

b) Up-to-date nomenclature of species names can be checked via http://www.bacterio.cict.fr/

c) Excluded are the yogurt starter cultures *Lactobacillus delbrueckii* subsp. *bulgaricus* and *Streptococcus thermophilus*.

d) Species not included in the 2009 updated EFSA list of biological agents intentionally added to food or feed recommended for qualified presumption of safety.

e) Probiotic properties have been documented in some strains of this species [205], but no applications for human use are currently known.

With the emphasis mainly on fermented dairy products, a steadily increasing range of yogurt-like products is available in the European market. Probiotic strains used in these products are generally, but not exclusively, derived from the gastrointestinal tract of presumed healthy humans. This is reflected by the high frequency in which strains of autochthonous *Lactobacillus* and *Bifidobacterium* species are applied in these products [14–17] (Table1). *Lactobacillus* is a member of the lactic acid bacteria and is naturally occurring on raw food and feed materials but is also a member of the intestinal tract of most mammals. Although also a lactic acid-producing bacterium, *Bifidobacterium* is not considered a typical member of the LAB, due to its remote phylogenetic position. To a lesser extent, also other LAB strains belonging to genera such as *Enterococcus*, *Lactococcus,* and *Pediococcus* are used as probiotics for humans mainly in dairy-based products. In contrast, strains of non-LAB bacteria such as *Bacillus*, *Propionibacterium,* and *Eschericha coli* used as probiotic are rarely included in dairy or other food commodities, but are usually applied as lyophilized or encapsulated pharmaceutical preparations. The use of yeasts in commercial probiotic products is virtually restricted to a single strain commonly referred to as *Saccharomyces cerevisiae* var. boulardii [18], although also other nonpathogenic yeast strains have been the subject of probiotic studies [19].

Importantly, not all microorganisms present in a probiotic product per definition have probiotic characteristics. A typical example is the case of strains of the yogurt starters *Lactobacillus delbrueckii* subsp. *bulgaricus* and *Streptococcus thermophilus*, which are applied mainly for technical reasons but are not commonly regarded as probiotics. The latter point is still under debate as literature searches have shown that viable yogurt starter cultures have the potential to improve lactose digestion and eliminate symptoms of lactose intolerance [20].

The relevance of reliable identification and typing of strains used in probiotic applications is still increasing because of the two main reasons. First, it is clear that the use of probiotic strains is no longer strictly restricted to food applications where history of safe use in (traditional) fermented foods was an important argument to neglect the importance of correct classification of probiotics. In recent years, however, probiotics are also more and more used in biotherapeutic and pharmaceutical applications, which are aimed at specific patient groups each with their own clinical risk profiles. As a part of the biosafety evaluation in the course of this type of clinical interventions, it is now commonly accepted that correct identification to the species level and even to the strain level is essential. Second, probiotic use no longer only involves monocultures but in the meanwhile has also witnessed the introduction of several mixed-strain formulations in food products with health-promoting claims as well as in therapeutic trials and applications. As part of any health claim dossier, it is clear that the individual strains composing such mixtures need to be fully characterized taxonomically.

### 2.2 Identification

At present, the inappropriate use of identification methods is regarded as the major cause of incorrect species designations of probiotic strains [21] and mislabeling of probiotic products [22–24]. Inconsistencies in the microbial identification of commercial products with probiotic claims affects their potential efficacy and safety record, and are likely to have a negative impact on consumer trust.

#### 2.2.1 Conventional phenotypic approaches for bacterial identification

For identification of probiotic bacterial strains, phenotypic tests or commercial miniaturized identification systems such as Analytical Profile Index tests are inadequate for species level resolution. In fact, it is recommended that biochemical characterization should not be used as a stand-alone approach for identification of any probiotic culture. Commercial identification systems may be useful to obtain a first tentative classification at the genus level in conjunction with primary phenotypic tests, but the identification result should in any case be confirmed by other (molecular) methods. It is important to highlight that the usefulness of (commercial) biochemical systems for identification of probiotic bacterial strains is limited, due to the high intraspecific phenotypic variability observed in many bacterial species, and due to the fact that updates of the identification databases linked to these systems are slow or even missing, are often incomplete, and that species entries are poorly documented without listing the reference strains used [25].

#### 2.2.2 Molecular approaches for bacterial identification

Despite the event of sequence-based approaches, DNA–DNA reassociation is currently considered as the “gold standard” for delineation and description of new bacterial species, but is impractical in routine identification of bacterial cultures. Other molecular methods are therefore preferred provided that they offer sufficient experimental reproducibility and a proper taxonomic resolution and that they make use of updated and easily available and validated identification databases. The use of molecular approaches for identification of species used in probiotic applications is covered in large detail in several review articles [14,15,26].

Sequence analysis of the partial or complete 16S ribosomal RNA (rRNA) gene is now commonplace as the first tool to use for the taxonomic positioning of probiotic cultures [27]. Next to the universal distribution of this gene in all bacterial species, which avoids the use of group-specific protocols, other obvious advantages of using this and other sequence-based methods include the high level of data reproducibility and exchangeability. Sequencing is now commonly outsourced to specialized service providers and sequencing centers, often with satisfactory to excellent results. The major bottleneck of the 16S rRNA gene sequencing approach for nonexperienced users, however, is the taxonomic interpretation of the sequence data by comparison with public sequence databases such as European Molecular Biology Laboratory (http://www.ebi.ac.uk/embl/) and GenBank (http://www.ncbi.nlm.nih.gov/genbank/). These databases often contain loads of unreliable, poorly documented, or incomplete sequence entries, which may compromise the accuracy of the identification result. Therefore, experienced users will have to make sure that only validated and complete 16S rRNA gene sequences are used for identification purposes and that full 16S rRNA gene sequences of multiple taxonomic reference strains per taxon (certainly including the type strain) are included in the identification database to encompass the genomic variation within the taxon. The Ribosomal Database Project (http://rdp.cme.msu.edu/) and the Silva rRNA database project (http://www.arb-silva.de/) are two free web-based applications that provide ribosome-related data and services to the scientific community, including online data analysis and aligned and annotated 16S rRNA gene sequences.

In addition to their use for straightforward identification of pure cultures, ribosomal sequences have also been exploited for culture-independent detection of probiotic microorganisms. The use of rRNA-targeted probes provides a unique insight into the dynamics of probiotic microorganisms in complex microbial communities. Nucleic acid probes have been designed to specifically target taxonomic groups at different levels of specificity (from genus to strain). The review by Ben Amor et al. [28] presents a good overview of the currently available and validated 16S rRNA-targeted oligonucleotide probes for the identification of LAB ranging from group and genus to species and subspecies level.

Despite its obvious advantages, it has been shown that 16S rDNA sequencing has a limited resolution for the discrimination of very closely related species, including those that are frequently used in probiotic foods and preparations. Within the genus *Lactobacillus*, for example, some species of the *L. delbrueckii* group, the *Lactobacillus casei* group, and the *Lactobacillus plantarum* group are difficult to separate even when using full 16S rRNA gene sequences [16,29]. In the case the probiotic strain belongs to one of these species, results of 16S rRNA gene sequence identification may only be reliable at the genus level and need to be complemented by other molecular methods to obtain classification at (sub)species level [21]. To this end, a series of DNA fingerprinting methods including repetitive DNA element (rep)-PCR, amplified fragment length polymorphism (AFLP), amplified ribosomal DNA restriction analysis, and tDNA intergenic spacer PCR have been evaluated and optimized for species identification of most probiotic microorganisms (Table2). In contrast to sequence-based approaches, however, fingerprint data are much less exchangeable and reproducible between laboratories and strongly rely on the availability of an extended and up-to-date database of reference profiles. For this reason, fingerprint-based identifications are best performed in expert labs that use standardized protocols and have access to in-house databases for identification of unknown cultures.

**Table 2:** DNA fingerprinting methods used for identification and/or typing of probiotic microorganisms

Method	LAB	*Bifidobacterium*	*Bacillus*	*Propionibacterium*	*Saccharomyces*
AFLP	[21,57]				
ARDRA	[206,207]	[211–213]		[219]	
ITS analysisa)			[217]	[220]	[68–70]
PFGE	[56–58,208]	[23,54,55]	[53]		[68]
RAPD	[52]		[53]		[68,69]
rep-PCRb)	[21,56,209,210]	[214–216]	[218]		[221,222]

a) Includes sequencing and/or restriction analysis of ribosomal ITS elements.

b) Targets repetitive elements such as (GTG)_5_, ERIC, and BOX for bacteria and M13 for yeasts.

The use of protein-encoding housekeeping genes essentially combines the technological advantages of 16S rRNA gene sequencing and the taxonomic resolution of many fingerprinting methods. Sequencing of one or preferably multiple of these genes as taxonomic markers is a crucial step forward in the development of standardized and globally accessible methods for the identification of probiotic cultures. For LAB, the combined sequence analysis of the *atpA, pheS*, and *rpoA* genes has been successfully explored for species identification of enterococci [30], lactobacilli [31], leuconostocs [32], and pediococci [33]. Analysis of partial *hsp60* [34] and *rpoB* [35] gene sequences have proven to be useful single-locus approaches for the differentiation of *Bifidobacterium* spp. However, concatenation of partial sequences of seven genes (*clpC*, *dnaB*, *dnaG*, *dnaJ1*, *purF*, *rpoC,* and *xfp*) may significantly increase the discriminatory power between bifidobacterial species [36]. Within the *Bacillus subtilis* group and related taxa, the housekeeping genes, *gyrB* [37] and *rpoB* [38], are often used for reliable species differentiation.

The most recent insights from whole-genome sequencing have indicated that even conserved genes such as protein-encoding housekeeping genes may be subjected to genomic rearrangements such as deletions, duplications, mutations, recombinations, and lateral gene transfer. This important finding thus suggests that the classification of microorganisms may not be accurately reflected by analyzing the sequences of one gene or a cluster of genes. The current availability of complete genome sequences of thousands of bacterial strains allows to investigate new avenues for bacterial phylogeny and identification. The average amino acid identity and the average nucleotide identity [39,40] are two parameters resulting from pairwise genome comparisons and averaging the sequence identities of shared orthologous genes (amino acid or nucleotide, respectively) that are already being used to compare whole-genome sequences for taxonomic purposes and, ultimately, to describe new bacterial species. Within the LAB and *Bifidobacterium* species used as probiotics for human applications, the complete genome sequences of more than 60 strains have been submitted to GenBank (Table3).

**Table 3:** List of complete genome sequences of *Bifidobacterium* and LAB species used as probiotics for human applicationsa),b)

Strainc)	GenBank Accession Number	NCBI RefSeq Numberd)
*Bf. adolescentis* ATCC 15703	AP009256.1	NC_008618.1
*Bf. animalis* subsp. *lactis* AD011	CP001213.1	NC_011835.1
*Bf. animalis* subsp. *lactis* B420	CP003497.1	NC_017866.1
*Bf. animalis* subsp. *lactis* BB-12e)	CP001853	
*Bf. animalis* subsp. *lactis* BLC1	CP003039.1	NC_017216.1
*Bf. animalis* subsp. *lactis* Bi-07	CP003498.1	NC_017867.1
*Bf. animalis* subsp. *lactis* B1–04	CP001515.1	NC_012814.1
*Bf. animalis* subsp. *lactis* CNCM I-2494	CP002915.1	NC_017215.1
*Bf. animalis* subsp. *lactis* DSM 10140	CP001606.1	NC_012815.1
*Bf. animalis* subsp. *lactis* V9	CP001892	
*Bf. bifidum* BGN4	CP001361.1	NC_017999.1
*Bf. bifidum* PRL2010	CP001840.1	NC_014638.1
*Bf. bifidum* S17	CP002220.1	NC_014616.1
*Bf. breve* ACS-071-V-Sch8b	CP002743.1	NC_017218.1
*Bf. breve* UCC2003	CP000303.1	
*Bf. longum* DJO10A	CP000605.1	NC_010816.1
*Bf. longum* NCC2705	AE014295.3	NC_004307.2
*Bf. longum* subsp. *infantis* ATCC 15697	CP001095.1	NC_011593.1
*Bf. longum* subsp. *infantis* 157F	AP010890.1	NC_015052.1
*Bf. longum* subsp. *longum* BBMN68	CP002286.1	NC_014656.1
*Bf. longum* subsp. *longum* F8	FP929034	
*Bf. longum* subsp. *longum* JCM 1217	AP010888.1	NC_015067.1
*Bf. longum* subsp. *longum* JDM301	CP002010.1	NC_014169.1
*Bf. longum* subsp. *longum* KACC 91563	CP002794.1	NC_017221.1
*Lb. acidophilus* 30SC	CP002559.1	NC_015214.1
*Lb. acidophilus* NCFM	CP000033.3	NC_006814.3
*Lb. amylovorus* GRL 1112	CP002338	
*Lb. amylovorus* GRL1118	CP002609.1	NC_017470.1
*Lb. casei* ATCC 334	CP000423.1	NC_008526.1
*Lb. casei* BD-II	CP002618.1	NC_017474.1
*Lb. casei* BL23	FM177140.1	NC_010999.1
*Lb. casei* LC2W	CP002616.1	NC_017473.1
*Lb. casei* str. Zhang	CP001084.1	NC_014334.1
*Lb. crispatus* ST1	FN692037.1	NC_014106.1
*Lb. delbrueckii* subsp. *bulgaricus* 2038	CP000156.1	NC_017469.1
*Lb. delbrueckii* subsp. *bulgaricus* ATCC 11842	CR954253.1	NC_008054.1
*Lb. delbrueckii* subsp*. bulgaricus* ATCC BAA-365	CP000412.1	NC_008529.1
*Lb. delbrueckii* subsp*. bulgaricus* ND02	CP002341.1	NC_014727.1
*Lb. fermentum* CECT 5716	CP002033	
*Lb. fermentum* IFO 3956	AP008937.1	NC_010610.1
*Lb. gasseri* ATCC 33323	CP000413.1	NC_008530.1
*Lb. johnsonii* DPC 6026	CP002464.1	NC_017477.1
*Lb. johnsonii* FI9785	FN298497.1	NC_013504.1
*Lb. johnsonii* NCC 533	AE017198.1	NC_005362.1
*Lb. plantarum* JDM1	CP001617.1	NC_012984.1
*Lb. plantarum* WCFS1	AL935263.1	NC_004567.1
*Lb. plantarum* subsp. *plantarum* ST-III	CP002222.1	NC_014554.1
*Lb. reuteri* DSM 20016	CP000705.1	NC_009513.1
*Lb. reuteri* JCM 1112	AP007281.1	NC_010609.1
*Lb. reuteri* SD2112	CP002844.1	NC_015697.1
*Lb. rhamnosus* ATCC 8530	CP003094.1	NC_017491.1
*Lb. rhamnosus* GGe)	FM179322.1	NC_013198.1
*Lb. rhamnosus* GGe)	AP011548	
*Lb. rhamnosus* Lc 705	FM179323.1	NC_013199.1
*Lb. salivarius* CECT 5713	CP002034	
*Lb. salivarius* UCC118	CP000233.1	NC_007929.1
*Lc. lactis* subsp. *cremoris* A76	CP003132.1	NC_017492.1
*Lc. lactis* subsp. *cremoris* MG1363	AM406671.1	NC_009004.1
*Lc. lactis* subsp. *cremoris* NZ9000	CP002094	
*Lc. lactis* subsp. *cremoris* SK11	CP000425.1	NC_008527.1
*Lc. lactis* subsp. *lactis* CV56	CP002365.1	NC_017486.1
*Lc. lactis* subsp. *lactis* Il1403	AE005176.1	NC_002662.1
*Lc. lactis* subsp. *lactis* KF147	CP001834.1	NC_013656.1
*Le. citreum* KM20	DQ489736.1	NC_010471.1
*Le. mesenteroides* subsp*. mesenteroides* ATCC 8293	CP000414.1	NC_008531.1
*Le. mesenteroides* subsp*. mesenteroides* J18	CP003101.1	NC_016805.1
*O. oeni* PSU-1	CP000411.1	NC_008528.1
*Pd. pentosaceus* ATCC 25745	CP000422.1	NC_008525.1
*S. thermophilus* CNRZ1066	CP000024.1	NC_006449.1
*S. thermophilus* JIM 8232	FR875178.1	NC_017581.1
*S. thermophilus* LMD-9	CP000419.1	NC_008532.1
*S. thermophilus* MN-ZLW-002	CP003499.1	NC_017927.1
*S. thermophilus* LMG 18311	CP000023.1	NC_006448.1
*S. thermophilus* ND03	CP002340	

a) Last updated on September 5, 2012.

b) Considering their importance in yogurt production, strains of *Lb. delbrueckii* subsp. *bulgaricus* and *S. thermophilus* were also included in the table.

c) The species name as mentioned in the Complete Microbial Genomes list of NCBI (http://www.ncbi.nlm.nih.gov/genomes/lproks.cgi) is not necessarily in accordance to the current bacterial nomenclature.

d) The Reference Sequence (RefSeq) database of NCBI provides a comprehensive standard dataset that represents sequence information for a species. RefSeq sequences are derived from GenBank records but differ in that each RefSeq is a synthesis of information, not an archived unit of primary research data.

e) Strains used as probiotics.

Finally, MS methods are becoming increasingly important for classification and identification of bacteria [41]. One of these methods, MALDI-TOF MS, allows to measure peptides and other compounds in the presence of salts and to analyze complex peptide mixtures which make it an ideal method for measuring nonpurified extracts and intact bacterial cells. The resultant MALDI-TOF MS spectra can be used to generate identification libraries for simple and high-throughput identification of unknown bacterial isolates. These reference libraries can either be constructed or provided on a commercial basis, such as the one integrated in the MALDI Biotyper system (Bruker Daltonics, Bremen, Germany), or can be generated on a local basis. Such local databases have been successfully used for the identification of *Lactococcus lactis* [42], the LAB genera *Leuconostoc*, *Fructobacillus,* and *Lactococcus* [43], LAB from traditional fermented foods [44], and the *Lb. casei* group [45]. Angelakis et al. [46] used a combination of commercial and local databases to successfully identify LAB and *Bifidobacterium* species present in probiotic drinks and yogurts.

#### 2.2.3 Yeast identification

Identification and classification of yeasts have traditionally been based on morphological, physiological, and biochemical traits. Various commercial biochemical kits have been developed for rapid yeast identification, but mostly for the clinical market. Currently, different molecular biology techniques are available for identification of yeasts.

A reliable starting point for the identification of probiotic yeasts is the D1/D2 large subunit rRNA gene sequence database, which encompasses virtually all known yeast species [47]. However, due to the fact that some distinct species show low-sequence divergence, the use of D1/D2 large subunit sequences is often complemented by PCR amplification of repetitive DNA elements [48] and by determination of additional gene sequences, such as the internal transcribed spacer (ITS) region of the rRNA gene cluster [49] or protein-coding genes such as the actin gene (*ACT1*; [50]), and the mitochondrial cytochrome oxidase 2 (*COX2*) and cobalamin-independent methionine synthase (*MET6*) genes [51].

### 2.3 Typing

#### 2.3.1 Bacteria

In many cases, it is highly important to characterize probiotic cultures at the individual strain level, a process generally referred to as typing. Such cases may include screening large microbiological collections for probiotic candidates, monitoring selected candidate strains during in vitro and in vivo trials, quality control of probiotic products, strain authentication in legal matters, and epidemiological surveys.

To a large extent, typing of probiotic microorganisms still relies on the use of molecular methods [15]. Several fingerprinting methods that have been used for species identification such as (rep)-PCR and AFLP are also suitable as typing methods up to the strain level (Table2). Especially in the case of AFLP, the flexibility in choice of specific restriction enzymes and selective PCR primers allows to test multiple combinations, which can significantly increase the discriminatory power at strain level. Depending on the number of isolates to be processed, the required speed of performance, and the expertise of the user, also randomly amplified polymorphic DNA (RAPD) and pulsed-field gel electrophoresis (PFGE) can be applied for typing of probiotic strains (Table2). RAPD allows fast and reliable comparison of larger sets of isolates in a single PCR run, but often lacks the same level of reproducibility offered by other PCR-based fingerprinting techniques, which is an important prerequisite for database construction. Nevertheless, RAPD is still frequently used to discriminate between probiotic strains [52,53]. Together with AFLP, by far the highest resolution at strain level can be achieved by PFGE. Although the latter method can be standardized, it requires a dedicated electrophoretic unit and considerable technical expertise. PFGE fingerprinting has been successfully used to differentiate or track probiotic strains in the genera *Bifidobacterium* [23,54,55], *Lactobacillus* [56–58], and *Bacillus* [59].

In addition to DNA fingerprinting, also sequence-based approaches have been used to discriminate or detect individual probiotic strains. The availability of complete genome sequences of probiotic strains not only broadens our biological knowledge of their functional potential, but also offers a myriad of new possibilities for strain differentiation. Based on internal nucleotide sequences of multiple (usually three to seven) housekeeping genes, several multilocus sequence typing (MLST) schemes have been developed for discrimination between bacterial isolates at the intraspecific level. For each gene, 450–500 bp internal fragments with a maximal number of polymorphisms are selected for amplification and subsequent gene sequence analysis. For each housekeeping gene, the different sequences present within a bacterial species are assigned as distinct alleles and, for each isolate, the alleles at each of the loci define the allelic profile or sequence type. The MLST method has several advantages, including reproducibility and data exchangeability and can be automated to a large extent. While MLST is primarily used to study population structure, evolution, and phylogeography of bacterial pathogens [60], depending on the species it also provides good discriminatory power to differentiate isolates for typing purposes. For bacteria, MLST schemes were first developed for clinically relevant microorganisms such as *Enterococcus faecium* (http://efaecium.mlst.net [61]) and *Bacillus cereus* (http://pubmlst.org/bcereus/ [62]). In recent years, MLST schemes have also been developed for several other species commonly applied as probiotics. MLST schemes relevant for typing of probiotic Lactobacilli include those developed for the species *Lb. casei* [63,64], *Lb. plantarum* [65], and *Lb. salivarius* [66]. Recently, an MLST scheme was made available for the probiotic *Bifidobacterium* species *Bf. animalis*, *Bf. bifidum*, *Bf. breve*, and *Bf. longum* [67]. Similar to the mlst.net database for bacterial pathogens, MLST sequence type databases have been launched, for example, for *Lb. casei* (http://www.pasteur. fr/recherche/genopole/PF8/mlst/Lcasei.html) and *Bifidobacterium* (http://www.pasteur.fr/recherche/genopole/PF8/mlst/Bifidobacterium.html). These public databases allow queries and downloads of allele sequences and allelic STs, provide several database tools and statistics, and are also open to additional data on novel strains and species.

In addition to MLST approaches, analysis of whole-genome sequences through comparative genomics can be very useful to identify unique gene sequences that allow to discriminate a given probiotic culture from other members of the same species. Such strain-specific target sequences can then be used to detect a given probiotic in complex environments, such as food matrices or fecal samples, without the need for culturing. Instead, culture-independent methods such as fluorescent in situ hybridization and real-time PCR are employed for direct detection and enumeration of the target strain in the sample.

#### 2.3.2 Yeasts

Typing of probiotic yeasts has largely been based on intraspecies polymorphisms located in the ITS regions of the ribosomal gene cluster. As an alternative to ITS sequencing, restriction fragment length polymorphism analysis of amplified ITS regions has been used to differentiate *Saccharomyces* yeasts isolated from various commercial probiotic and biotherapeutic products claimed to contain *S. cerevisiae* or “*S. boulardii*” [68]. In the latter study, the ITS-based approach was combined with RAPD and PFGE fingerprinting to differentiate *S. cerevisiae* var. boulardii strains from other strains in the species. Discrimination of individual strains within *S. cerevisiae* is unlikely with a single typing technique [69]. In a comparative study with ITS-based approaches, microsatellite DNA polymorphism analysis and retrotransposon Ty917 hybridization analysis were considered to offer the highest discriminatory power to distinguish probiotic and clinical *S. cerevisiae* var. boulardii isolates from other clinical *S. cerevisiae* isolates [70]. More recent developments in typing of *S. cerevisiae* strains focus on the design of MLST schemes. So far, the MLST approach has mainly been applied in wine yeast typing but does not seem to offer the same level of resolution as microsatellite analysis [71].

## 3 Safety assessment

### 3.1 Introduction

In general, the microorganisms used in the production of food fermentation have a long history of safe use and are often referred to as “food grade” or GRAS (Generally Recognized As Safe) microorganisms [14]. Hence, this is the case for most LAB and bifidobacteria [72–74]. In Europe, the qualified presumption of safety (QPS) concept exists with a list of microorganisms that can be considered safe for use. The microorganisms intended for human use are regulated in the EU in the context of novel food regulation (Regulation (EC) No. 258/97; http://ec.europa.eu/food/food/biotechnology/novelfood/index_en.htm). For microorganisms (probiotics) used as additives in animal feed (Regulation (EC) No. 1831/2003), official guidelines for their safety assessment have been fixed at EU level by Regulation (EC) No. 429/2008.

In recognition of the importance of assuring their safety for human purposes, even among LAB that are generally considered to have a very good safety record, new and existing probiotic strains need to be characterized with respect to a number of safety aspects. Epidemiological surveillance studies are very important to evaluate the risk of probiotics used in a specific population. In general, four types of side effects of probiotics can be distinguished: systemic infections, metabolic and enzymatic effects, immunomodulation and adjuvants, and gene transfer. Among these, the most direct safety risk associated with the consumption of microorganisms is infection (e.g. endocarditis), especially in immunocompromised individuals. In general, the risk of infection by probiotic *Lactobacillus* or *Bifidobacterium* strains is similar to the risk of infection by commensal strains, and thus products containing such probiotic strains present a negligible risk to consumers [75]. On the other hand, some strains of other LAB groups such as enterococci are agents of opportunistic infections. Rare cases of infection associated with certain *Lactobacillus* strains have been reported, mostly in immunocompromised patients [76]. Bifidobacteria are extremely rarely associated with infections [77]. *Bifidobacterium*-associated infections are most likely caused by bifidobacteria from the patients’ own microbiota [78,79]. A recent study reported that the administration of the probiotic *E. coli* strain Nissle 1917 to immunocompromised patients may lead to severe adverse effects [80].

To establish safety guidelines for probiotic microorganisms, an FAO/WHO working group recommended that probiotic strains are characterized by a series of tests including antibiotic resistance, metabolic activities, toxin production, hemolytic activities, infectivity in immunocompromised animal models, side effects in humans, and adverse outcomes in consumers [10].

Although still under debate, it is generally agreed that probiotic safety aspects should cover the isolation history and species identity of the probiotic culture and provide phenotypic and/or genotypic evidence showing that the culture does not harbor acquired antibiotic resistance traits, putative virulence factors, or other pathogenic properties [25,81,82]. In addition, it has been suggested that translocation, adhesion, and colonization may also contribute to the safety dossier of a probiotic strain. However, current problems in method standardization and data interpretation may need to be solved first before the usefulness of these parameters can be truly accepted.

### 3.2 Isolation history and taxonomic characterization

Although still a matter of debate, it has been suggested that probiotic strains should originate from the species of intended use. One can argue that a probiotic strain originating from the gastrointestinal tract of a healthy human can function better in a similar environment from where it was originally isolated. Although this point of view has been supported by the fact that most current successful strains are indeed of human origin, some animal-derived strains have also shown positive effects on humans.

In addition to documenting its strain history, the first step in (a) microorganism(s) or product being referred to as a probiotic is to identify the microorganism using internationally accepted methodologies, preferably by combining phenotypic and genotypic methods. The practical approaches for accurate determination of species identity of probiotic cultures have been discussed extensively elsewhere in this report. Correct identification at species level also implies the use of scientifically recognized names and adequate designation of particular strains. The current state of evidence suggests that different strains can possess different features related to different safety risks, implying that it is not possible to identify the specific safety risks associated with a probiotic strain without proper identification. Reliable identification is also necessary to avoid the inclusion of pathogenic microorganisms in probiotic products.

### 3.3 Antimicrobial resistance

Due to the indiscriminate use of antibiotics in human and veterinary medicine and as animal growth promoters, antibiotic resistance has become an increasingly common characteristic in (food borne) microorganisms [83], causing serious problems in treatment of microbial infections. Antibiotic resistance in bacteria may be intrinsic or acquired. Intrinsic resistance is a naturally occurring trait that may be characteristic for a given species or genus, whereas acquired resistance derives either from genetic mutations or acquisition of foreign DNA from other bacteria. Probiotic strains with nontransmissible antibiotic resistances do not usually confer a safety concern. To some extent, nontransmissible antibiotic resistance might even be a useful property, if the probiotic strain is to be used as a prophylactic agent in the treatment of antibiotic-associated diarrhea [84,85]. However, antibiotic resistance linked to transferable plasmids and mobile elements is a different case because of the possibility of resistance spreading to other, potentially more harmful bacteria. In the end, such increases in the dissemination of antibiotic resistance genes can drastically reduce the therapeutic possibilities in infectious diseases. Given the documented presence of antibiotic resistance genes in LAB and the indirect evidence that these genes could be transferred along the food chain [86–88], it is therefore relevant to assess the presence of transferable antibiotic resistances in LAB strains that are or shall be used as probiotics for human consumption, an opinion which is adopted by EFSA [89]. Different expert panels have indicated that strains harboring transferable antibiotic resistance genes are not suitable for use as probiotics [25,90]. In this context, the specific risks related to each probiotic strain must be carefully identified.

For phenotypic antimicrobial susceptibility testing of bacterial species with clinical relevance such as enterococci, *enterobacteria* and certain *Bacillus* species, expert committees such as those appointed by the Clinical and Laboratory Standards Institute (CLSI; http://www.clsi.org/) and the British Society for Antimicrobial Therapy (BSAC; http://www.bsac.org.uk/) have proposed a series of standardized and harmonized methods that can be easily performed in most routine labs. However, for nonenterococcal LAB and bifidobacteria, which together account for the vast majority of probiotic cultures currently used, no such standards are available from CLSI, BSAC, or other committees. As a result, a variety of noncongruent methods and protocols for the determination of antibiotic susceptibilities of nonenterococcal LAB have been reported in the literature using agar (overlay) disc diffusion, E-test, broth dilution, and agar dilution [91]. In general, dilution methods and E-test are preferred as reference methods over conventional diffusion-based tests, as the former techniques allow determining minimal inhibitory concentration (MIC) values that provide a more reliable indication of the intrinsic or acquired nature of a given resistance phenotype. However, due to the fact that many nonenterococcal LAB require specific growth conditions in terms of medium acidity and carbohydrate supplementation, conventional media such as Mueller–Hinton (as recommended by CLSI) and Iso–Sensitest agar (as recommended by BSAC) are not suitable for susceptibility testing of probiotic lactobacilli, pediococci, lactococci, and bifidobacteria. In the course of the EU project PROSAFE, two new test medium formulations have been developed for antimicrobial susceptibility testing of nonenterococcal LAB [91]. The resulting LAB susceptibility test medium (LSM) consists of a mixture of Iso-Sensitest broth (90% v/v) and de Man-Rogosa-Sharpe (MRS) broth (10% v/v), supplemented with 0.3 g/L *l*-cysteine hydrochloride for bifidobacteria. The use of the LSM formulation has further been substantiated in another EU project, which is Assessment and Critical Evaluation of Antibiotic Resistance Transferability in the Food Chain (EU-FP6 project) (http://www.aceart.net), which resulted in the proposal to use this medium as a standard medium for susceptibility testing of lactobacilli and bifidobacteria from food and nonfood origin. Recently, the intra and interlaboratory performance of the LSM medium and related formulations, specifically adapted for MIC testing of bifidobacteria, *S. thermophilus* and *L. lactis*, has been positively evaluated in a small-scale harmonization study [92]. In parallel, a standard operating procedure, based on data from the PROSAFE and Assessment and Critical Evaluation of Antibiotic Resistance Transferability in the Food Chain (EU-FP6) projects, has been validated as an official shared standard of ISO/IDF (International Dairy Federation), i.e. ISO 10932:2010 (IDF 223:2010) standard “*Milk and Milk Products – Determination of the Minimal Inhibitory Concentration (MIC) of Antibiotics Applicable to Bifidobacteria and Non-Enterococcal Lactic Acid Bacteria*” (http://www.iso.org/iso/iso_catalogue/catalogue_tc/catalogue_detail.htm?csnumber=46434).

The interpretation of MIC data in order to decide whether a (potential) probiotic strain harbors an acquired or atypical antibiotic resistance trait highly depends on the availability of epidemiological cut-off values. The European Committee on Antimicrobial Susceptibility Testing (http://www.eucast.org) has proposed a number of definitions toward a uniform MIC interpretation. A microorganism is either defined as a wild-type (WT) or nonwild-type (NWT) member of a species by the absence or presence, respectively, of acquired and mutational resistance mechanisms to the antimicrobial agent in question. The strain in question is categorized as WT or NWT within a species based on the cut-off values appropriate for that species and determined with a defined phenotypic test system. This cut-off value will not be altered by changing circumstances and WT microorganisms may or may not respond clinically to antimicrobial treatment (http://www.srga.org/eucastwt/eucastdefinitions.htm). Ideally, cut-off values are defined at the species level, not at the genus level. In this respect, the availability of correct species identification is an important prerequisite to define such species-specific cut-off levels. For a range of *Lactobacillus* species or species groups, including *Lb. delbrueckii* group, *Lb. plantarum*, *Lb. sakei*, *Lb. rhamnosus*, *Lb. paracasei* group, *Lb. reuteri,* and *Lb. fermentum*, specific MIC distributions and tentative cut-off values for a range of antibiotics have been proposed [93]. Importantly, these data were obtained using a standard protocol that is largely compatible with the experimental procedures outlined in ISO 10932:2010 (IDF 223:2010). Likewise, MIC distribution data for several important bifidobacterial taxa, including *Bf. adolescentis* group, *Bf. longum* group, *Bf. bifidum*, *Bf. catenulatum* group, and *Bf. animalis*, have been published [94].

Probiotic cultures belonging to the NWT population of a species and thus in which phenotypic resistance has been detected may have acquired antibiotic resistance genes located on plasmids or (conjugative) transposons that may be further transmitted to other microorganisms. The other possibility, i.e. that the antibiotic resistance is due to mutations of chromosomal housekeeping genes, represents a neglectable risk of horizontal dissemination. Verification of the presence of antibiotic resistance genes is thus crucial and can be achieved by the use of dedicated PCR assays when a very specific resistance trait is targeted. The majority of acquired resistance genes so far found in (probiotic) LAB and bifidobacteria are those conferring resistance to tetracycline (i.e. *tet* genes) and erythromycin (i.e. *erm* genes). PCR primers specific for *tet* and *erm* genes are published and have been validated for use in *Lactobacillus* and *Bifidobacterium* [95,96]. In the case that the potential presence of multiple, rare, or silent (i.e. not phenotypically detectable) resistance genes needs to be verified, the use of dedicated microarrays [97,98] can be recommended.

If the presence of one or multiple resistance genes is verified, the next step in the risk safety assessment could be to check transfer of the gene(s) under experimental conditions [87,99]. However, failure to demonstrate in vitro horizontal gene transfer does not exclude the risk of dissemination of genes. Thus, while negative transfer experiments do not provide evidence for the absence of transfer, standardized transfer methods are still required to estimate whether the probability for resistance transfer is low or high. A standardized conjugation protocol has been developed and validated by several laboratories to asses antibiotic resistance transfer between lactococcal species [100]. However, such protocols still need to be established for in vitro transfer experiments with lactobacilli and bifidobacteria.

### 3.4 Virulence and pathogenic properties

In general, potential probiotic strains should be screened in vitro for their interactions with cell lines to investigate possible cytotoxic or cytopathological effects after growth in different media, for the presence of known virulence genes (e.g. lecithinase activity, toxin genes) and for the presence of mobile genetic elements. After these in vitro tests for potentially safe use, in vivo toxicity tests and persistence studies would be required. A recent study by Hütt et al. [101] shows how to evaluate the in vivo safety and persistence of *Lactobacillus* strains in the gastrointestinal tract of healthy adult volunteers after oral consumption of high doses of lactobacilli.

The long history of safe use of probiotic LAB is the best evidence for the safety of probiotic products. *Lactococcus* and *Lactobacillus* are most commonly given the “generally recognized as safe” or GRAS status. Other LAB genera like *Streptococcus* and *Enterococcus* and other genera that could be used as probiotics contain opportunistic pathogens [102]. The absence of pathogenicity of any potential probiotic strain must be shown to prove its safety. It should be addressed that in general the number of infections with *Lactobacillus* strains are very low. For instance, the risk of *Lactobacillus* infections is estimated at about one case per 10 million people over a century of probiotic consumption in France [103]. Even after performing different clinical trials and human studies, including one involving enteral feeding of premature infants with a commercial *Lactobacillus rhamnosus* strain called GG (title of the strain derived from the names of Goldin and Gorbach), no pathogenic potential could be indicated [104]. However, a recent review [76] reported all cases of lactobacillemia identified through a Medline search of articles published between 1950 and 2003. From the 241 cases identified, lactobacilli were implicated in endocarditis, bacteremia, and localized and other infections. *Lactobacillus casei* and *Lb. rhamnosus* (among which a case due to consumption of large quantities of fermented milk with the probiotic strain GG in the months before hospitalization; [105]) were the species most commonly responsible for these pathologies. In recent years, some cases of liver and spleen abscesses caused by lactobacilli have been described [106–108]. Most of these lactobacillemia cases are associated within the population with a reduced immune function. In a recent comprehensive review on probiotic infections in patients receiving probiotics in conjunction with nutritional support, it appeared that all 20 case reports of adverse events in 32 patients involved infections due to *Lb. rhamnosus GG* or *Saccharomyces boulardii*; the risk factors included central venous catheters and disorders associated with increased bacterial translocation [109]. The dominance of these two probiotic species in these reported cases may be linked to their wider use in clinical settings rather than their increased virulence. The question if food containing probiotics is at the origin of the endocarditis cases is not clear because *Lactobacillus* isolates from blood cultures and from commercial dairy products are different based on the carbohydrate fermentation [110]. Another report described the case of a person suffering from a *Lactobacillus*-associated endocarditis after having teeth extracted due to caries. The nongastrointestinal way of consumption is maybe at the origin of this infection [111]. In general, it could be said that the frequency of *Lactobacillus* infective endocarditis is very low, between 0.05 and 0.4% of the total of bacterial endocarditis cases. The results should be handled with care due to false or incomplete identification of the strains in some studies, which highlighted the necessity of molecular tools for strain identification [112].

In contrast to *Lactobacillus* spp., the genus *Enterococcus* has a higher potential safety risk, although enterococci are used as starter cultures in the food industry as well as probiotics [73,113], it is emerging as a major cause of nosocomial infection causing endocarditis, bacteremia, central nervous system infections, neonatal infections, urinary tract infections and other infections, and its isolates are increasingly found to carry virulence factors [114]). The origin of enterococcal pathogenicity is linked to factors involved in adhesion, translocation, and immune evasion [115]. A multiplex PCR can be used for the detection of the presence of specific enterococcal virulence genes such as *asa*1, *gel*E, *cyl*A, *esp*, and *hly* [116]. As an example, none of the 12 probiotic *E. faecium* strains tested by this multiplex PCR tested positive for the virulence genes. This multiplex PCR can be used for testing the intrinsic virulence capacity of a strain and, together with other molecular typing methods, it could be an additional criterion in assessing the safety of new potential probiotic *E. faecium* isolates [25]. *Enterococcus faecalis* carries a pathogenicity island of 153 kb containing several virulence factors and is, therefore, believed to be more virulent than *E. faecium*, although horizontal transfer of the entire pathogenicity island into the chromosome of *E. faecium* has been demonstrated [117,118]. *Enterococcus faecium* is mostly used as probiotic like in four commercial tablet products in Japan, although they are mislabeled as containing *E. faecalis* [119].

Probiotic *Bacillus* strains are gaining increasing interest because their spore-forming capacity has obvious advantages in relation to stability and viability of the probiotic product when this contains spores instead of vegetative cells [17]. Moreover, it has become apparent in recent years that several *Bacillus* species, such as *B. subtilis*, have adapted to a lifestyle in the gastrointestinal tract [120]. The use of *Bacillus* spores as probiotic implies the direct consumption of high concentrations of viable cells. One of the commercial products produced in Europe and marketed in at least three EU countries (a.o. Belgium), namely Bactisubtil, seems to contain *B. cereus* spores, although the product label originally mentioned *B. subtilis*. The same strain as in Bactisubtil has also been used in the animal feed product Paciflor, which has been withdrawn in 2002 from production due to the ability of the strain to produce diarrhea enterotoxins hemolysin BL (from *B. cereus*) (hemolytic enterotoxin complex) and nonhemolytic enterotoxin. Two other human probiotics produced outside Europe (Biosubtyl and Subtyl in Vietnam and Biovicerin in Brazil) also contain *B. cereus* spores. Strains belonging to the *B. cereus* group are known to be able to cause two kinds of food-borne illness (Belgian Superior Health Council, 2009). First, there is an emetic (vomiting) illness due to the ingestion of food containing the heat-stable toxin cereulide. The second is a diarrheal infection due to the ingestion of *B. cereus* strains producing heat-labile enterotoxins in the small intestine. Several published PCRs for these virulence genes or toxins could be used to screen *B. cereus* group strains [121]. Detection of toxin genes in *B. cereus* allows assessment of the enterotoxic potential of an isolate, but not necessarily of its enteropathogenicity. Cytotoxicity tests can be used as an approximate estimation of the latter in the absence of other valid tests. Nevertheless, the detection of the presence of toxin genes by PCR-based approaches remains important for strain characterization of *B. cereus*. Probiotic products can also contain strains of, for example, *B. licheniformis, B. clausii, B. subtilis,* and *B. pumilus*. Although much less important in food-poisoning incidents, strains of several of these *Bacillus* species are also known to be potential producers of heat-stable or heat-labile toxins [122]. Because the *B. cereus* toxin PCRs are specific for this species and not valid for detection of toxins in other *Bacillus* species, at present only a cytotoxicity test can be performed to exclude any pathogenic opportunity of the *Bacillus* strain in question [122].

*Eschericha coli* Nissle strain 1917 is a well-known probiotic strain used in clinical trials to treat ulcerative colitis [123]. Yet, treatment with this strain has also been associated with sepsis such as a case of severe sepsis in a preterm infant [124]. Using a mice model, the safety of *E. coli* Nissle strain 1917 was assessed under different conditions of defective immunity and intestinal microbiota [80]. It was found that if both the microbiota and adaptive immunity are defective, the strain may have potentially severe adverse effects. For other *E. coli* strains that would be considered as potential probiotics, it is important to ascertain that they do not belong to one of the pathogenic *E. coli* groups, such as Shiga-toxin-producing, enterohaemorrhagic, enterotoxigenic, enteroinvasive, enteropathogenic *E. coli*, etc. [125]. Several multiplex PCRs are described to investigate if an *E. coli* strain contains virulence genes and belongs to one of these pathogenic groups [126].

Propionibacteria and bifidobacteria belong both to the coryneform bacteria and are used in (probiotic) dairy products. The safety of these bacteria as dairy microorganisms has been recently reviewed [126]. In contrast to the cutaneous *Propionibacterium* spp. or the so-called “acnes group”, the dairy *Propionibacterium* spp. are regarded as safe and do not carry any known virulence factor, although *P. thoenii* and *P. jensenii* strains show β-hemolytic activity. *Bifidobacterium* is among the safest genera used as probiotics and the risks of healthy consumers being seriously infected by eating dairy products containing bifidobacteria are extremely low. Nevertheless, as bifidobacteria are common members of the human intestinal microbiota, they may behave as opportunistic pathogens like other commensal bacteria and indeed some commensal bifidobacteria have been connected with certain dental infections, pulmonary infections, bacteremia, abscesses, and bloodstream infections. The nonprobiotic *B. dentium* is the only bifidobacterial species classified as a pathogen.

*Saccharomyces cerevisiae* var. boulardii (present in Enterol®) has been associated with cases of fungemia [128]. Several factors constitute excessive and undue risk for development of *Saccharomyces* fungemia during probiotic administration. These factors are the patient’s immunocompromised state during critical illness, the potential for live yeast spore contamination by the healthcare workers’ hands during preparation of the probiotic capsule or sachet for administration, and introduction of live yeast from contaminated hands (even after hand washing) to catheter sites [129].

Currently, probiotics or synbiotics are being used experimentally in patients hospitalized in intensive care units, so-called critically ill patients, because in these patients significant alterations in the gut microbiota occur that can lead to severe sepsis with associated multisystem organ dysfunction and death. It remains uncertain whether the use of probiotics or synbiotics in these circumstances is beneficial or even dangerous to the clinical outcome. There is a need for well-designed multicenter studies with a defined mixture of bacteria on a defined group of critically ill patients before any conclusion can be drawn. In addition, the end-points have to be clearly defined [130]. Recently, it has been concluded from a clinical trial of probiotic prophylaxis in predicted severe acute pancreatitis patients, that probiotic prophylaxis should not be administered in this category of patients because the probiotic preparation used (Ecologic® 641 consisting of six strains of viable and freeze-dried bacteria, namely *Lactobacillus acidophilus, Lb. casei, Lactobacillus salivarius, L. lactis, Bifidobacterium bifidum*, and *Bifidobacterium lactis* in a total daily dose of 10^10^ bacteria) did not reduce the risk of infectious complications and was even associated with an increased risk of mortality [131]. Following the publication of this report, several reactions of colleagues appeared among which probiotics should only be contemplated if the integrity of the gastrointestinal tract is not severely compromised [132]. In a recent comprehensive review on probiotic infections in patients receiving probiotics in conjunction with nutritional support, it appeared that only 3 out of 53 trials, in which 4131 patients received probiotics, showed increased complications, which were largely noninfectious in nature and in specific patient groups (e.g. transplant and pancreatitis) [109]. The authors recommend preliminary safety trials when a probiotic is to be investigated for the first time in a patient group receiving nutritional support and that caution should be taken in patients with risk factors for adverse events.

With the advent of next-generation sequencing techniques, the number of full bacterial genomes being sequenced has rapidly increased. In Table3, >60 complete genomes of bifidobacterial and LAB species used as probiotics are listed, and >100 more genomes are being sequenced. Comparative genomics are now being used to find core genes, niche-specific genes, and genes linked to specific probiotic traits, but can as well be used to find or exclude virulence or antibiotic resistance genes or to find indications of chromosomal integration of horizontally acquired DNA (e.g. sequence context screening of t_RNA_ genes), which could indicate the potential of horizontal transfer of virulence or resistance genes. For example, from a comparative genomic analysis between a probiotic and a clinical *E. faecalis* strain, it seems that several of the above–mentioned enterococcal virulence factors present are absent in the probiotic strain *E. faecalis* Symbioflor I [133]. Likewise, a genomic comparison between the probiotic strain *E. coli* Nissle 1917 and other *E. coli* strains has indicated the lack of defined virulence factors (i.e. α-hemolysin, P-fimbrial adhesions) in the probiotic strain [134], but another study indicated that genetic variations (e.g. mutations) and gene expression differences, rather than genomic content of virulence genes per se, contribute to the divergence in the pathogenic traits between *E. coli* strains [135].

### 3.5 Metabolic activities associated with production of toxic substances

Another requisite of probiotics is that the probiotic bacteria should not produce harmful substances by metabolic activities. One way to test this is to assess whether the strain converts food components or biological secretions into secondary substances harmful to the host. For example, some intestinal bacteria are known to convert proteins and their digested products into ammonia, indol, phenols, and biogenic amines (histamine, tyramine, putrescine, etc.) [136]. There are no real indications on the production of harmful compounds by *Lactobacillus* and *Bifidobacterium* species. Araya-Kojima et al. [137,138] measured the enzyme activities related to the consumption and generation of ammonia in *Bifidobacterium* species of human origin. Compared with other bacteria of the intestinal microbiota, *Bifidobacterium* species have a lower deaminase activity involved in the production of ammonia from amino acids but a higher ammonia assimilation activity. Secondary bile acids are important harmful substances that are produced by intestinal bacterial actions on body secretions. They may exhibit carcinogenicity by acting on the mucous-secreting cells and promoting their proliferation, or they may act as promoters of carcinogenesis [139]. Many intestinal bacteria, including *Bifidobacterium* and *Lactobacillus* species, can deconjugate conjugated bile acids [140]. However, *Bifidobacterium* spp., *Lactobacillus* spp., *Leuconostoc lactis* subsp. *lactis* and *S. thermophilus* have been reported to lack the 7α-dehydroxylase activity that is related to the production of secondary bile acids [141,142]. For *Enterococcus*, cytolytic substance and other virulence factors have been reported [143].

### 3.6 Platelet-aggregating activity and mucus degradation activity

Platelet-aggregating activity has been considered to be a required test in the assessment of probiotic safety. Aggregation of platelets by bacteria is thought to contribute to the progression of infective endocarditis [144]. In this context, it has been reported that platelet-aggregating activities of *Lb. rhamnosus* and *Lb. paracasei* subsp. *paracasei* isolated from infective endocarditis, laboratory strains of the same species, and strains of the species *Lb. acidophilus*, *Lb. fermentum*, *Lb. oris*, *Lb. plantarum*, and *Lb. salivarius* are very strain specific [145]. The aggregation is thought to be associated with the proteins on the outer cell layer. The properties of the outer cell layer have been measured by hydrophobicity, hydroxyapatite adhesion, and salivary aggregation. *Lactobacillus rhamnosus* strains isolated from infective endocarditis have higher activities than do laboratory strains of *Lb. rhamnosus* [146]. As for the other virulence factors, the activities of glycosidases and proteases (arylamidase), which might enable the breakdown of human glycoproteins and the synthesis and lysis of human fibrin clots, have been measured in *Lb. rhamnosus, Lb. paracasei* subsp. *paracasei*, and other strains. Some strains produce these enzymes, suggesting that they may have an infective property in causing endocarditis [147]. However, a study that aimed to measure the enzymatic activities relating to degradation of intestinal mucus glycoprotein in several strains of *Lactobacillus* and *Bifidobacterium* found no such activity in these strains [148]. Further research on the structure of the outer cell layer or the above-mentioned enzyme activities in probiotic bacterial strains is expected. Whether the outer layer structure, which contains surface proteins, glycoproteins, and lectins, is really related to infectivity and whether glycosidases, proteases (arylamidase), and other enzymes capable of degrading human intestinal cells are related to infection remain to be elucidated.

### 3.7 Safety parameters “under debate”: colonization, adhesion, and translocation

As mentioned above, the QPS list used in Europe describes the microorganisms that can be considered safe for use in food applications. Members on this list often have “a long history of safe use”, without any negative effect mentioned in the literature. The QPS list is based on identification at the species level, while it is generally accepted that probiotic effects are strain specific. It is fair to say that also virulence factors are strain specific and may vary largely within a species. As an example, *E. coli* is often mentioned. This species comprises harmless commensal strains, which we all carry with us without any problem, but pathogenic strains which are, for instance, enteroinvasive *E. coli* (http://www.fda.gov/Food/Food Safety/FoodborneIllness/FoodborneIllnessFoodbornePatho gensNaturalToxins/BadBugBook/ucm071298.htm) or en-terohemorrhagic *E. coli* (http://www.fda.gov/food/foodsafety/foodborneillness/foodborneillnessfoodbornepathogensnatur altoxins/badbugbook/ucm071284.htm) are widely known and studied. Underlying mechanisms for invasiveness and pathogenicity are gradually being understood, although many uncertainties remain.

In the process of infection and invasion, three aspects may be important: a first contact or adhesion between the bacterial strains and the epithelial cells, a further translocation of the strain into or through the intestinal epithelium and proliferation in the sterile body site, either at the level of the lamina propria, the mesenteric lymph nodes, or systemically [149,150], leading to sepsis, endocarditis, and bacteremia [151]. For this reason, it has been suggested that the adhesion and translocation potential of a strain should be considered as part of the safety evaluation of probiotics [152].

Adhesion has been considered as a possible probiotic characteristic, since adhesion can prolong the intestinal colonization of the probiotic strain, improve the beneficial immune interactions, occupy receptors that could otherwise also “accommodate” pathogens and fortify the intestinal barrier. Clearly, the subject is controversial and further research into the many factors that may influence the outcome of adhesion tests is required. Different tests, different epithelial cell lines, whether or not mucus was included, or simply different laboratories all seem to result in different findings. In addition, also the physiological status of the bacteria seems to matter and, consequently, many doubts exist on the value of in vitro tests to predict the in vivo effect of a particular strain. Adhesion may also depend on the local competition with the commensal microbiota, on the number of bacteria consumed, and above all, eventual translocation will heavily depend on the immune status of the hosts. Effects of translocated bacteria may also be very different. The way to deal with this is far from clear given that the absence of pathogenicity and infectivity is a prerequisite of probiotic safety. The isolation of LAB or bifidobacteria from clinical cases, however, is likely to be the result of opportunistic infections [153–155]. To some extent, the increasing isolation rate from these infections may be due to an increased awareness of the role of these bacteria in opportunistic infections, or may result from the use of improved identification methods. A sensible approach in studying this would be to consider the question whether invasion of the host by the bacteria leads to infection and whether infection results in a severe outcome [156].

Translocation is probably a much more important phenomenon than adhesion, as adhesion without translocation is rarely a problem and could, as argued above, even be beneficial. Systemic infection by intestinal bacteria after translocation is a cause of opportunistic infection in immunocompromised hosts [157,158] and may be linked to intestinal mucosal injury, immunodeficiency of the host, or bacterial overgrowth [156,159,160]. “Infective” bacterial translocation from the intestine is difficult to induce in healthy animals [161 162], although “controlled” bacterial translocation can be seen as a highly regulated, physiological event that occurs continuously in healthy subjects at a low rate [162]. When the integrity of the intestinal barrier is disturbed or when the immune system is not able to confine an infection, pathogenic or commensal bacteria can reach the bloodstream and cause septicemia [163]. Therefore, antibiotic treatment, administration of immunosuppressive agents, or induction of colitis are used to measure bacterial translocation [152,162,164]. While bacterial translocation does not occur easily in healthy specific pathogen-free animals, it is known to occur for a long duration in germ-free mice [156,165,166], linked to the immature intestinal barrier and the underdeveloped immune system of these germ-free animals [167,168].

In conclusion, assessment of the safety of probiotics from various angles is not a simple task [156]. The test item that has been attracting attention is whether the bacteria possess infectivity. Assessment of the ability to cause opportunistic infection is difficult. The acute and chronic toxicity tests probably provide circumstantial evidence. However, observations of the passage of bacteria across the intestinal barrier and invasion of the host body by translocation provide more direct data for determining infectivity.

### 3.8 Genetically modified microorganisms (GMMs)

Alongside natural bacterial and yeast strains, GMMs have been designed to treat specific diseases by targeting specific pathogens and/or toxins, or through the delivery of immunosuppressive proteins (reviewed in [169]). The use of these so-called designer probiotics in humans effectively means that GMM are deliberately released into the environment, which calls for specific measures and monitoring to guarantee their safety. Such measures include well-documented evidence that probiotic GMM (i) do not possess antibiotic selection markers, (ii) cannot accumulate in the environment, and (iii) cannot transfer their genetic modification(s) to other (micro)organisms [170]. The most challenging of these is the prevention of environmental accumulation that can be achieved through active and/or passive biological containment. Active containment is achieved by the production of a compound toxic to the GMM under regulation and control of an environmentally responsive element, whereas passive containment relies on complementation of an auxotrophy by supplementation with either an intact gene or the essential metabolite (for review see [171]). The latter method was applied in the design of a recombinant interleukin-10-producing *L. lactis* strain for treatment of Crohn’s disease patients [172]. This recombinant is deficient for the thymidylate synthase gene (*thyA*), which is essential for growth of *Lc. lactis*, and thus holds a negligible risk to accumulate and spread in the environment in the absence of thymine or thymidine.

## 4 Quality control of final products

### 4.1 Microbial contamination of the final product

Probiotic foods have steadily gained popularity over the past decades and a wide variety of foods nowadays contain probiotic cultures. Strains are selected based on their specific health-promoting effects, but it is obvious that the safety aspects are also carefully considered to ensure that they do not pose any health risk for the consumer. The safety evaluation usually includes screening for (transferable) antibiotic resistance genes, virulence or pathogenic properties, undesirable metabolic activities, and collection of evidence to demonstrate a “history of safe use”, etc. [82]. Once a strain has been approved, it must still be determined whether the analytical methods that are normally used to verify the microbiological safety of the ingredients and the finished products can also be used for the product with the probiotic strains. For microbiological methods, this is not very obvious.

Finished products typically contain ca. 7 to 8 log colony-forming unit (CFU) per gram and the initial starter may contain more than 11 log CFU per gram, so it should be taken into account that the presence of undesirable contaminants is masked by the abundance of the beneficial microorganisms. Theoretically, this problem is most likely to occur with nonselective cultural methods. However, even with selective methods, this possibility should be considered, in particular because many of these methods include a nonselective preenrichment step. This is, for instance, the case for various internationally recognized protocols, for example, for the isolation and detection of *Salmonella* (ISO standard 6579:2002). Joosten et al. [173] clearly demonstrated that false-negative results could be obtained for the detection of *Salmonella* in infant powder with probiotics. The high incidence of false-negative results can probably be explained by the metabolic activities, properties of the probiotic bacteria, in particular the production of organic acids, whose accumulation could render the preenrichment broth into a hostile environment for *Salmonella*. By using double-strength buffered peptone water supplemented with vancomycin (10 μg/mL) and malachite green (100 μg/mL) and nonfat dry milk powder (10 g/L) as preenrichment medium, the recovery of *Salmonella* was much better [173]. For the detection of other pathogenic bacteria, especially when a nonselective enrichment is used, a similar problem could be observed. By adding antimicrobial compounds to selectively suppress the growth and/or metabolic activity of the probiotic bacteria, this problem could be resolved, but still more research is needed to optimize such enrichment procedures. For the enumeration of the total microbiota, especially the contaminating microbiota (without the enumeration of the probiotic strain), the addition of 2% sodium phosphate or 5% glycerophosphate could solve the problem [174]. To study more in detail the problem of detection of contaminants in starter cultures and probiotics, a specific ISO working group started in 2010 (ISO/TC 34/SC9).

### 4.2 Microbial composition

Several research labs have independently analyzed the microbial composition of probiotic dairy products [14,23,24,175–177]. The overall conclusion from these studies is that a considerable number of dried food supplements and-–although to a much lesser extent–-dairy products are incorrectly or inadequately labeled, with regard to the correct identity of the incorporated probiotic strains. At a time when consumers become more and more aware of the important link between nutrition and health, it is thus of paramount importance that probiotic products with claimed health-promoting effects are well controlled regarding their microbial composition. Ideally, microbial analysis of probiotic products relies on standardized and accurate procedures for both the isolation and the identification of the implemented strains. In practice, however, microbial quality control of probiotic products is carried out using a culture-dependent or a culture-independent approach, or a combination of both, with each of these approaches having specific drawbacks and limitations. Especially in the case of multispecies products, a reliable taxonomic inventory may be difficult to achieve.

In a culture-dependent approach, the most crucial step is the recovery of the probiotic strain(s) from the product matrix, using one or more selective isolation media [178,179]. Some probiotic strains are the result of isolation campaigns that employed very specific growth conditions that are not always provided by commercial media or by the standard set of incubation parameters used for isolation. In this context, it has been shown that especially *Bifidobacterium* strains may be hard to recover from the original products, and that selective isolation of bifidobacteria from products that also contain LAB is not always straightforward [23,177]. Although still far from optimal, improved protocols and selective media with a higher performance have been proposed for specific enumeration and isolation of bifidobacteria from (probiotic) samples [23,180]. After isolation, a limited number of colonies are usually subjected to taxonomic characterization for the purpose of species identification as described above. When the most optimal isolation conditions are applied but culture-dependent analyses are negative, it can be concluded that the targeted bacteria are either absent or are present in numbers beneath 1000 CFU/mL or CFU/g, which is generally considered to be the detection limit of culture-dependent analyses.

In order to circumvent the possible pitfalls of conventional culturing, culture-independent methods are increasingly being used for both qualitative and quantitative microbial analysis of probiotic products, because it may overcome some of the major disadvantages or limitations of culture-dependent approaches in terms of speed, taxonomic resolution, and reproducibility, Denaturing gradient gel electrophoresis (DGGE) is a DNA fingerprinting technique that has been widely used to unravel the microbiological composition of commercial probiotic products [23,24,181–183]. Essentially, the DGGE method relies on (i) the isolation of total genomic DNA from the probiotic product, (ii) the selective amplification of a hypervariable region of the 16S rRNA gene by community PCR, and (iii) the electrophoretic separation of the resulting pool of PCR amplicons based on sequence content using DGGE [184]. In theory, the resulting DGGE fingerprint will visualize all species present in a given probiotic product by a unique band, which can be identified by band position analysis, band sequencing, and/or band hybridization. Despite the simplicity of its methodological concept, however, the interpretation of a DGGE fingerprint requires specific expertise, as some band positions may coincide with those of other phylogenetically closely related bacteria, some species may be represented by multiple bands due to the so-called 16S rDNA multi-operon effect, or species present in numbers beyond the detection level may not be visible [184]. To improve the detection capacity of DGGE, group-specific primers can be used [184].

Although DGGE has been successfully used to determine the microbiological composition of probiotic products, it does not provide a lot of information regarding the relative number of each species present. As discussed elsewhere in this report, a range of validated 16S rRNA-targeted oligonucleotide probes is available for the identification of LAB ranging from group and genus to species and subspecies level [28]. Commonly, these probe sequences are converted to genus-, species- or strain-specific PCR primers in the design of real-time quantitative PCR assays to assess the relative concentration of the constituting strains and species in a given probiotic product [185–187].

### 4.3 Microbial concentration and stability

The quality of a microbiological product, such as a starter or more generally a powder enriched with microbial cells, is generally quantified by its content in microbial cells, and among these cells two properties being expected: the ability to divide and to display a metabolic activity when introduced in a medium or a food composition. These properties are generally represented by a term called viability. However, recent studies have shown that cell division and metabolic activity are not always correlated, since microbial cells are able to reach a transient state where no cell division occurs but metabolic activity is maintained [188], leading to a distinction between viability (or ability to divide) and vitality (related to the metabolic activity). These observations have led to the distinction between three classes: viable and cultivable (VC), nonviable (NV), and viable but non cultivable (VBNC). This last class has been the subject of intensive studies followed by the development of a dedicated method in order to make the distinction between VC, NV, and VBNC states [189]. The correct assignment of a probiotic product to one of these classes is crucial, given the fact that the FAO/WHO definition of probiotics [7] explicitly excludes NV formulations [190]. According to this definition, dead cells of (beneficial) bacteria that are shown to trigger or modify biological responses such as anti-inflammatory effects [191] should thus not be named probiotics. However, even for quality-checked probiotic products in which the active strains are considered to be in a VC state, it has been shown that a part of the bacterial population may become VBNC during storage [192,193]. While the technological and economical importance of this finding is clear, possible effects of VC-to-VBNC transitions on probiotic efficacy remain to be determined.

Traditional microbiological methods rely on direct counting of microbial cells either by microscopy or by plate counting. The second method is generally preferred, since it gives directly the amount of cells able to divide with a result being expressed as “colony-forming units”. However, this method presents several drawbacks: it is labor intensive, it is subjected to variability, and it is not able to display the VBNC state. The emergence of fluorescence-based techniques has led to a great improvement of the understanding of microbial viability and its evolution throughout processes [194]. The use of specific fluorescent dyes allows to get a strong insight into the level of the intracellular structures in relation with cell viability. The most used techniques involve stains displaying different behaviors related to membrane permeability and/or degree of damage. The combination of fluorescent-based techniques with high-throughput experimental techniques has led to new opportunities in order to improve the analytical tools dedicated to the determination of the cellular properties [195]. In this area, flow cytometry is an emerging technology in the field of food, environmental, and industrial biotechnology for the assessment of microbial viability [196]. The main advantage of this method is that it does not require a cultivation step of the microorganisms before analysis. Indeed, microorganisms present in the sample are separated and aligned cell by cell in an optical analysis chamber by a flow-focusing method. In this way, optical properties can be acquired at the single-cell level allowing to take into account the intrinsic heterogeneity of the microbial population (heterogeneity has been observed even in a clonal population). In addition to the above-mentioned advantages, a flow cytometry-based method can be automated in order to follow cell properties on-line during a process. It can also be applied to follow product quality during storage after downstream processing steps, such as freeze drying and spray drying [197].

### 4.4 Strain authenticity and genetic stability

The authenticity of a probiotic culture is an important criterion to ensure the expected quality and thus predicted health-promoting effect of the probiotic product. Linked to strain authenticity is genetic stability, which reflects the susceptibility of the culture concerning genomic rearrangements in the course of its natural evolution. These rearrangements may reflect small variations introduced at specific or random positions of the genome through point mutations, deletions, and insertions, but may also be linked to larger structural variations resulting from homologous recombination (vertical inheritance) and horizontal gene transfer events. Although this is a highly relevant issue in order to ensure that specific health-promoting characteristics and functionalities are not affected during long-term preservation and production, few studies have reported comprehensive data documenting the genetic stability of commonly used probiotic strains. Ideally, a rigorous assessment of a strain’s genetic stability requires the availability of its whole-genome sequence. In the absence of this information, however, the use of molecular typing techniques probably provides the best estimate of genetic stability at the individual strain level.

A preliminary verification of bacterial strain authenticity can be obtained by partial 16S rRNA gene sequencing. If this analysis confirms the species identity of the probiotic culture, further characterization at strain level may be required. Elsewhere in this report, a series of typing methods is discussed which could be used for this purpose. However, as different typing methods offer different levels of genotypic resolution, the choice of the method and the appropriate protocol are crucial. As far as DNA fingerprinting approaches are concerned, AFLP analysis and PFGE of macrorestriction fragments offer the highest resolution at strain level. These two methods are excellent tools for genotypic comparisons throughout the production or shelf-life period of a product. Sequence-based approaches such as the MLST schemes developed for several *Lactobacillus* and *Bifidobacterium* species are also relevant for typing of probiotic cultures, although their usefulness in strain authenticity matters still needs to be verified. Although not supported by producers and distributors of commercial probiotic products, deposit of probiotic cultures into an internationally recognized culture collection would provide an enduring source of reference material for confirmation of genetic stability.

Although it is generally assumed that *Lactobacillus* and *Bifidobacterium* strains used in probiotic applications show a high genotypic stability under different production conditions, more direct and in-depth evidence for this hypothesis is needed. Ideally, this should be done using a series of different molecular typing methods or, ultimately, comparative genomics. A number of genome projects have revealed that species used in probiotic products have specifically adapted themselves to specific environments. This is exemplified in the genomes of *Bifidobacterium longum* subsp. *longum* and *Bf. longum* subsp. *infantis* by the presence of many genes involved in the uptake and utilization of host-indigestible complex carbohydrates and polyols, supporting their genome adaptation to the human large intestine, where these are the predominant nutrient sources [198,199]. The genome of *L. delbrueckii* subsp. *bulgaricus*, which is applied worldwide in yogurt production, is in a phase of rapid reductive evolution, as a possible result of its adaptation from a plant-associated habitat to a milk environment through the loss of superfluous functions and protocooperation with *S. thermophilus* [200]. In the same way, comparative genomics could also determine the potential of probiotic strains to acquire and/or transfer DNA elements during host passage and long-term colonization, which is a research area that has remained virtually unexplored.

### 4.5 Labeling and claims

Official controls by national authorities are performed to ensure verification of compliance with food law. Apart from the risk of using unauthorized strains, product mislabeling is a known problem, partly because of the use of phenotyping or genotyping methods with a lack of discriminative power [21]. In addition to official controls, private controls by food-producing companies are important in the frame of protection of patented strains and industrial property rights.

In their “Guidelines for the Evaluation of Probiotics in Food” document, the FAO/WHO working group [10] recommends that the following information should be described on the label of probiotic products:

1. genus, species, and strain designation. Strain designation (definition) should not mislead consumers about the functionality of the strain;
2. minimum viable numbers of each probiotic strain at the end of the shelf-life;
3. the suggested serving size must deliver the effective dose of probiotics related to the health claim;
4. health claim(s);
5. proper storage conditions;
6. corporate contact details for consumer information.

In most countries, only general health claims are currently allowed on foods containing probiotics. The FAO/WHO working group [10] recommended that specific health claims on foods be allowed relating to the use of probiotics, where sufficient scientific evidence is available. Such specific health claims should be permitted on the label and promotional material. For example, a specific claim that states that a probiotic “reduces the incidence and severity of rotavirus diarrhea in infants” would be more informative to the consumer than a general claim that states “improves gut health”. It is recommended that it has to be the responsibility of the product manufacturer that an independent third party review by scientific experts in the field be conducted to establish that health claims are truthful and not misleading. In line with the suggestions of the FAO/WHO working group [10], in 2006 the European Parliament and the Council published a novel regulation (Regulation (EC) No. 1924/2006) on “Nutrition and Health Claims Made on Foods”) [12]. This regulation applies to all nutritional and health claims relating to all types of food intended for final consumers, thus also including probiotic products brought to the market with a health claim. The regulation aims to harmonize the nutrition and health claims at European level in order to better protect consumers, including commercial communications (labeling, presentation, and promotional campaigns) and trademarks and other brand names that may be construed as nutrition or health claims. The regulation establishes the authorization procedures required to ensure that claims on food labeling, presentation, and advertising are clear, concise, and based on evidence accepted by the whole scientific community.

## 5 Concluding remarks

The first objective of Working Group “8651 Probiotics” of the Belgian Superior Health Council was to review the state of knowledge as regards to the methodologies for the microbiological characterization of strains and products with purported probiotic activity. Above all, this advisory report illustrates that characterization of probiotics in terms of taxonomic identity, biological safety, and product quality is a challenging task that requires a multidisciplinary approach. In addition, it also helps to reinforce current or even identify new research opportunities. For instance, the ongoing integration and translation of massive (meta)genomic datasets into knowledge and tools that allow more accurate determination of a strain’s identity, a better demonstration of its safety and more powerful prediction of its functionalities are still in their infancy, but will have important implications in the development of new probiotic formulations as well as a better mechanistic understanding of the existing ones [201,202]. For characterization and quality control of multistrain and multispecies formulations especially, high-throughput pyrosequencing approaches are expected to become a standard soon. Also, little is known about the impact of food format and food ingredients on the survival, physiology, and efficacy of the incorporated probiotic strain(s) [203,204]. Such information is essential to determine and predict possible differences in the behavior of a strain in pure culture or in a food product, but also to challenge the degree of flexibility of regulatory standards and recommendations in the context of bioequivalence.

The few examples mentioned above clearly indicate that probiotics are one of the key areas in food research where academics, industry, regulatory agencies, health professionals, and policy makers are destined to communicate and collaborate in the interest of consumer and patient. This advisory report hopes to provide a benchmark for ongoing and future developments in the microbial characterization of probiotics, and will in due course be followed by a second report of Belgian Superior Health Council Working Group 8651 focusing on functional properties and health prospects of probiotics.

## Abbreviations

AFLP: amplified fragment length polymorphism
BSAC: British Society for Antimicrobial Chemotherapy
CFU: colony-forming unit
CLSI: Clinical and Laboratory Standards Institute
DGGE: denaturing gradient gel electrophoresis
EFSA: European Food Safety Authority
FAO: Food and Agriculture Organization
GG (*L. rhamnosus* GG): designation of the strain derived from the names of Goldin and Gorbach
GMMs: genetically modified microorganisms
IDF: International Dairy Federation
ITS: internal transcribed spacer
LSM: LAB susceptibility test medium
MIC: minimum inhibitory concentration
MLST: multilocus sequence typing
NDA: Panel on Dietetic Products, Nutrition and Allergies of the EFSA
NV: nonviable
NWT: nonwild-type (member of a species)
QPS: qualified presumption of safety
PFGE: pulsed-field gel electrophoresis
RAPD: randomly amplified polymorphic DNA
rRNA: ribosomal RNA
VBNC: viable but noncultivable
VC: viable and cultivable
WHO: World Health Organization
WT: wild-type (member of a species)
